# Elimination and Eradication of Neglected Tropical Diseases with Mass Drug Administrations: A Survey of Experts

**DOI:** 10.1371/journal.pntd.0002562

**Published:** 2013-12-05

**Authors:** Jeremy D. Keenan, Peter J. Hotez, Abdou Amza, Nicole E. Stoller, Bruce D. Gaynor, Travis C. Porco, Thomas M. Lietman

**Affiliations:** 1 Francis I Proctor Foundation, University of California, San Francisco, San Francisco, California, United States of America; 2 Department of Ophthalmology, University of California, San Francisco, San Francisco, California, United States of America; 3 Texas Children's Hospital Center for Vaccine Development, Baylor College of Medicine, Houston, Texas, United States of America; 4 National School of Tropical Medicine, Baylor College of Medicine, Houston, Texas, United States of America; 5 Sabin Vaccine Institute, Washington, D.C., United States of America; 6 Université Abdou Moumouni de Niamey, Niamey, Niger; 7 Programme National de Lutte Contre la Cécité, Niamey, Niger; 8 Department of Epidemiology & Biostatistics, University of California, San Francisco, San Francisco, California, United States of America; 9 Institute for Global Health, University of California, San Francisco, San Francisco, California, United States of America; Imperial College London, Faculty of Medicine, School of Public Health, United Kingdom

## Abstract

**Background:**

Lymphatic filariasis, onchocerciasis, schistosomiasis, soil-transmitted helminths, and trachoma are the five most prevalent neglected tropical diseases in the world, and each is frequently treated with mass drug administrations. We performed a survey of neglected tropical diseases experts to elicit their opinions on the role of mass drug administrations for the elimination of these infections.

**Methodology/Principal Findings:**

We sent an online survey to corresponding authors who had published an article about a neglected tropical disease from 2007 to 2011. Of 825 unique authors who were invited to complete the survey, 365 (44.2%) responded, including 234 (28.4%) who answered questions regarding one of the five most prevalent neglected tropical diseases. Respondents had varying opinions about the goals of programmatic activities for their chosen neglected tropical disease, with elimination or eradication identified as the most important goal by 87% of lymphatic filariasis respondents, 66% of onchocerciasis respondents, 55% of trachoma respondents, 24% of schistosomiasis respondents, and 21% of soil-transmitted helminth respondents. Mass drug administrations, other non-medication health measures, and education were generally thought to be more important for elimination than vector control, development of a new tool, or the presence of a secular trend. Drug resistance was thought to be a major limitation of mass drug administrations for all five neglected tropical diseases. Over half of respondents for lymphatic filariasis and trachoma thought that repeated mass drug administrations could eliminate infection within ten years of the initiation of mass treatments.

**Conclusions/Significance:**

Respondents for lymphatic filariasis, onchocerciasis, and trachoma were more enthusiastic about the prospects of elimination and eradication than were respondents for schistosomiasis or soil-transmitted helminths. Mass drug administrations were generally believed to be among the most important factors for the success of elimination efforts for each of the five neglected tropical diseases, highlighting the opportunity for integrating drug distributions.

## Author Summary

Mass drug administrations are used for each of the five most common neglected tropical diseases: lymphatic filariasis, onchocerciasis, schistosomiasis, soil-transmitted helminths, and trachoma. Three of these infections—lymphatic filariasis, onchocerciasis, and trachoma—are officially targeted for elimination, and mass drug administrations play a key role in the elimination plans for each. While progress has been demonstrated for each of these diseases, it is unclear whether researchers of these diseases think that elimination is feasible, or whether mass drug administrations should play an important role given the potential for drug resistance. We performed a survey of neglected tropical diseases experts to assess their opinions on the likelihood of elimination and the role of mass drug administrations for the five most common neglected tropical diseases. Most experts in lymphatic filariasis, onchocerciasis, and trachoma thought elimination was the appropriate goal of treatment programs, whereas most experts in schistosomiasis and soil-transmitted helminths thought that treatment programs were intended to control, but not eliminate, infection. Drug resistance was thought to be a major limitation for each of the infections. Although there were differences between the five infections, mass drug administrations, non-medication health measures, and education were generally thought to be the most important pieces of a control program.

## Introduction

The most prevalent neglected tropical diseases (NTDs), including lymphatic filariasis (LF), onchocerciasis, schistosomiasis, soil-transmitted helminths (STHs), and trachoma, are routinely treated with periodic mass drug administrations (MDAs) [1], [2]. Repeated mass drug treatments may progressively reduce the burden of infectious agent in the entire community, resulting in a form of herd protection [3]. MDAs may even allow for elimination or eradication of one or more of these high-prevalence NTDs [4]. The World Health Organization (WHO) has called for elimination of LF and trachoma by 2020, which was recently endorsed in the London Declaration on Neglected Tropical Diseases. The United States Agency for International Development (USAID) aims to eliminate onchocerciasis from the Americas by 2016, and the African Programme for Onchocerciasis Control (APOC) has recently begun shifting its goals in Africa from control towards elimination [5]. The strategic plans for elimination for each of these NTDs includes MDAs.

The concepts control, elimination, and eradication have been refined over the past few decades [6], [7]. Eradication requires the reduction of the incidence of an infection to zero worldwide, while elimination refers to the reduction of the incidence of infection to zero in a defined geographic area. Some have referred to elimination of an infectious disease as interrupting the transmission of infection. Control refers to the reduction of the infection to a locally acceptable level. Elimination and control require continued interventions to prevent re-transmission and re-emergence of the infection, whereas eradication does not. Note that despite the classic definitions described above, the phrase “elimination of disease as a public health problem” is also used; this concept of elimination would not require zero incidence of infection.

Although the possibility of disease elimination or eradication is frequently discussed, it is unclear whether infectious disease experts believe these are feasible goals. In this study, we performed a survey of NTD experts to elicit their opinions on the likelihood of elimination or eradication for the five most prevalent NTDs.

## Methods

### Ethics statement

We obtained ethical approval from the University of California, San Francisco Committee on Human Research. The research adhered to the tenets of the Declaration of Helsinki.

### Study population

We emailed an invitation for the survey to corresponding authors who had recently published research or a commentary on an NTD, with an emphasis on those authors who studied a disease traditionally treated with MDAs. To identify corresponding authors, we performed a search on PubMed for articles published between January 2006 and March 2011 in *Annals of Internal Medicine, BMJ, Clinical Infectious Diseases, Emerging Infectious Diseases, Journal of Infectious Diseases, Journal of the American Medical Association, Lancet*, *New England Journal of Medicine, and PLoS Medicine* using the following search terms: antihelminthic, *Ascaris*, *Brugia*, filariasis, helminthiasis, hookworm, leishmaniasis, leprosy, *Necator*, neglected diseases, *Onchocerca*, onchocerciasis, roundworm, *Schistosoma*, schistosomiasis, soil-transmitted helminths, trachoma, *Trichuris*, tropical diseases, trypanosomiasis, whipworm, and *Wuchereria*. We excluded papers that dealt solely with the basic science of the organisms. In addition, we identified the corresponding author(s) from every article published in *PLoS Neglected Tropical Diseases* since the inception of the journal (October 2007 to March 2011). We reviewed each article for the email of the corresponding author(s).

### Survey

We used surveymonkey.com (Palo Alto, CA) to administer the survey, with an initial invitation sent by email in February 2011 and a reminder email sent three weeks later. We designed the survey to elicit opinions about elimination and eradication of five NTDs that are typically treated with MDAs: LF, onchocerciasis, schistosomiasis, STHs, and trachoma (Text S1). Specific definitions for elimination and eradication were emphasized in the survey and listed at the top of the screen for all survey questions. Specifically, we defined control as “reduction of infection to an acceptable level; requires continued intervention,” local elimination as “reduction of infection to zero in a defined geographical area; requires continued measures to prevent re-establishment of transmission,” and global eradication as “permanent reduction of infection to zero worldwide, not requiring any further intervention.” Survey respondents were asked to identify the NTD about which they were most knowledgeable, and whether MDAs played a role for treatment of this disease. If the respondent thought that MDAs did not play a role for this disease, they were asked to identify an NTD for which MDAs did play a role, and to answer the remaining questions about that disease.

### Statistical analysis

We assessed for differences in the distribution of responses between five NTDs of interest (LF, onchocerciasis, schistosomiasis, STHs, and trachoma), treating the survey responses as the outcome and the five-level NTD variable as the predictor. We used a chi square test for nominal outcomes and ordered logistic regression for ordinal outcomes (i.e., control vs. elimination vs. eradication, and time to elimination/eradication). The ordinal logistic regression models did not violate the proportional odds assumption according to the Brant test. For a question in which respondents were asked to rank six factors in order of importance for elimination, any of the factors that were unranked were assigned the average of the remaining non-ranked factors. We performed all analyses in Stata 12.0 (Statacorp, College Station, TX).

## Results

We extracted 902 unique email addresses from articles on NTDs published from January 2006 to March 2011, and were able to successfully send an invitation to 856 email addresses, which corresponded to 825 unique authors. Of these, 365 (44.2%) responded to the survey, including 234 (28.4%) who answered questions about one of the five NTDs of interest. In total, we analyzed 98 responses for schistosomiasis, 55 for STHs, 32 for LF, 27 for onchocerciasis, and 22 for trachoma. Respondents generally held a doctorate degree, with 63.7% having a PhD, 16.4% an MD, and 15.9% an MD, PhD; Table S1. Respondents worked in a wide variety of locations, including Africa (49.3%), South America (25.9%), East Asia (20.9%), and South Asia (11.4%); see Table S1.

**Figure 1:**
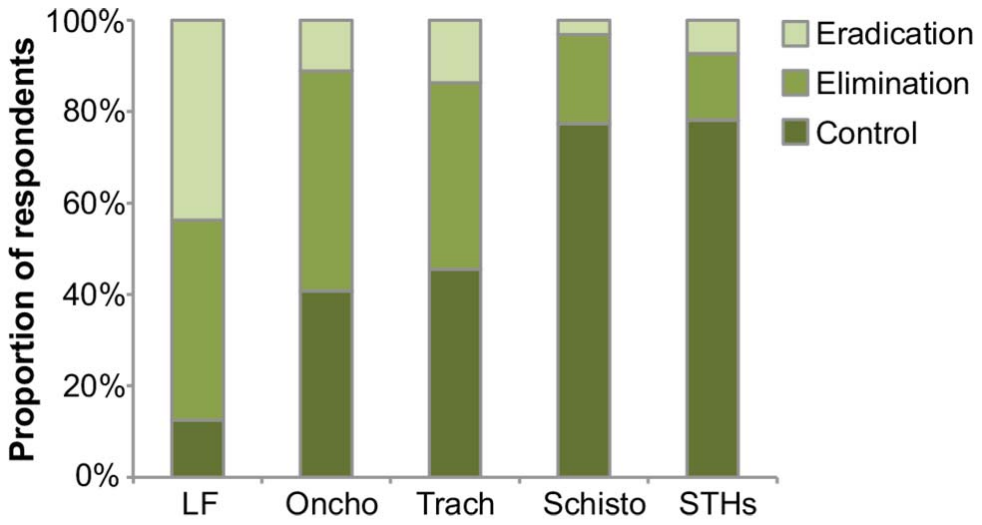
Opinions about the goal of treatment programs for five neglected tropical diseases. Eradication was defined as the permanent reduction of infection to zero worldwide, not requiring any further intervention. Elimination was defined as reduction of infection to zero in a defined geographical area, requiring continued measures to prevent re-establishment of transmission. Control was defined as reduction of infection to an acceptable level, which requires continued intervention. LF = lymphatic filariasis, Oncho = onchocerciasis, Trach = trachoma, Schisto = schistosomiasis, STHs = soil-transmitted helminths.

We asked about the primary treatment goal for the selected NTD, offering control, local elimination, or global eradication as options. Responses differed between the five NTDs of interest; for example, nearly half of LF respondents believed eradication to be the primary goal, whereas greater than 75% of schistosomiasis and STH respondents thought control was the primary goal (Figure 1 and Table S1; *P*<0.001).

### Elimination

**Figure 2:**
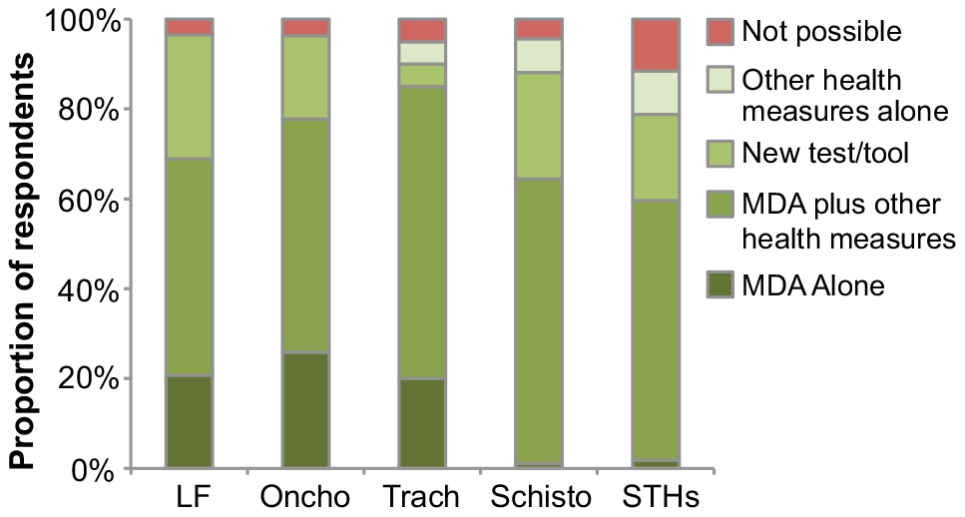
Circumstances under which local elimination of infection could occur for each of five neglected tropical diseases. Respondents were asked under what conditions elimination could occur: mass drug administrations (MDAs) using currently available drugs, MDAs plus other health measures, other health measures alone, or only if a new diagnostic test or interventional tool were developed. Alternatively, respondents could answer that elimination was not possible under any circumstances. LF = lymphatic filariasis, Oncho = onchocerciasis, Trach = trachoma, Schisto = schistosomiasis, STHs = soil-transmitted helminths.

Figure 2 shows the conditions that survey respondents thought sufficient to result in local elimination. More than half of respondents for each of the five NTDs believed MDAs to be necessary for local elimination. Although most respondents believed that in the absence of other health programs MDAs could not lead to local elimination, approximately 20–25% of LF respondents, trachoma respondents, and onchocerciasis respondents thought that use of MDAs alone could be sufficient for elimination (Figure 2 and Table S1; *P*<0.001 comparing distribution of responses for five NTDs).

**Figure 3:**
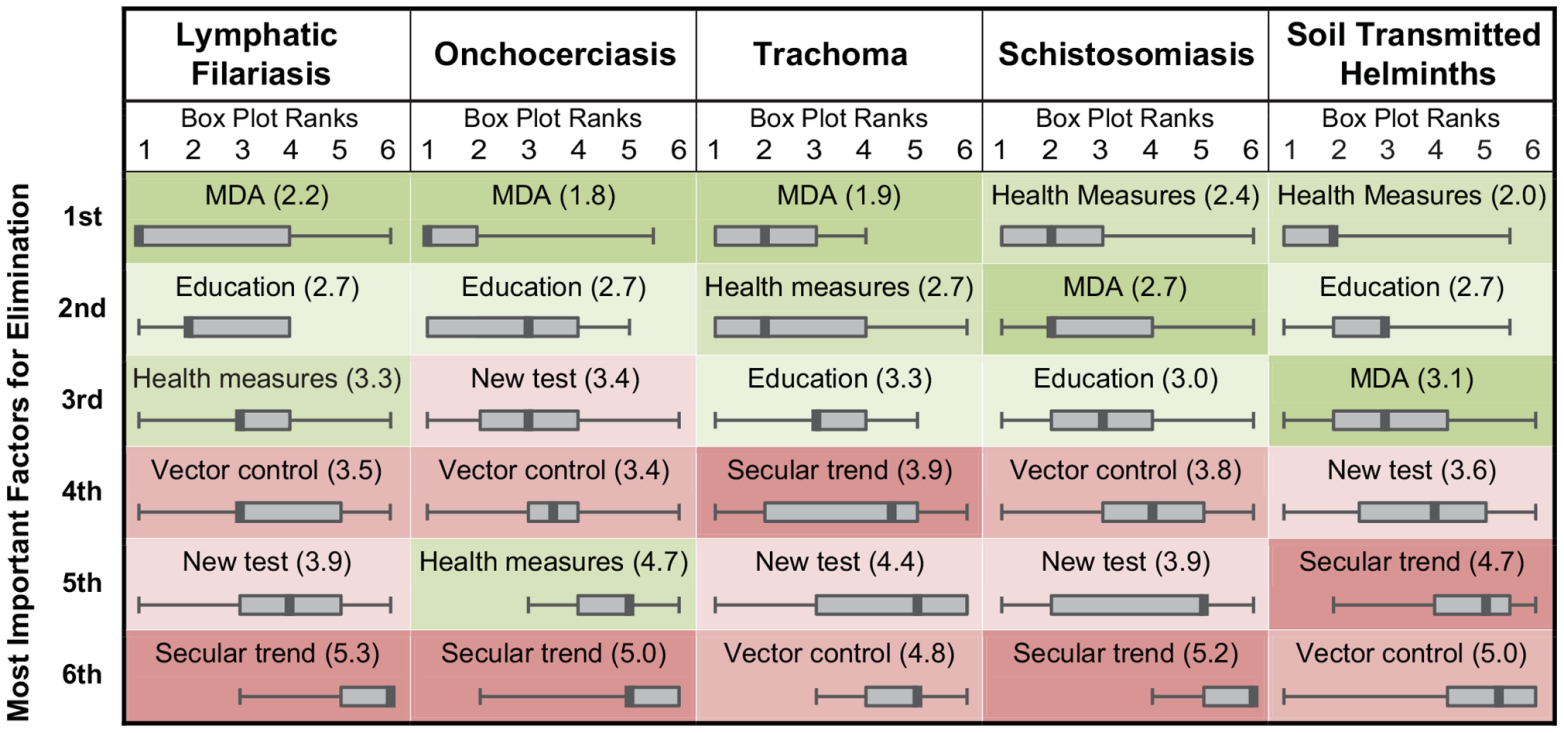
Ranking of most important factors for local elimination for five neglected tropical diseases. Each column lists the factors ranked for a specific disease, with the most important factor listed on top. In each cell, the mean rank for each factor is shown in parentheses, and the distribution of the ranks are shown in a box plot with the median rank shown as a thick grey line, and the range of rankings shown with whiskers. The boxes are color coded by that factor's mean rank across all five diseases, from most important (darkest green) to least important (darkest red).

Respondents ranked several factors in order of importance for elimination. Figure 3 lists each of these factors in order from most important to least important for each of the five targeted NTDs. Also shown is the distribution of the rankings given for each factor, represented as a box plot. The rankings varied for each of the five NTDs, though MDAs, health measures (such as sanitation improvements), and community education were generally ranked highest and the development of a new test/tool and presence of a secular trend were generally ranked lowest. Since each factor is color-coded in Figure 3, this general agreement can be observed graphically as a clustering of the green cells at the top of the figure and red cells at the bottom. The box plots for MDAs show a narrower distribution for onchocerciasis and trachoma than for the other diseases, suggesting higher agreement among the respondents of these two infections that MDAs are the most important factor for elimination.

**Figure 4:**
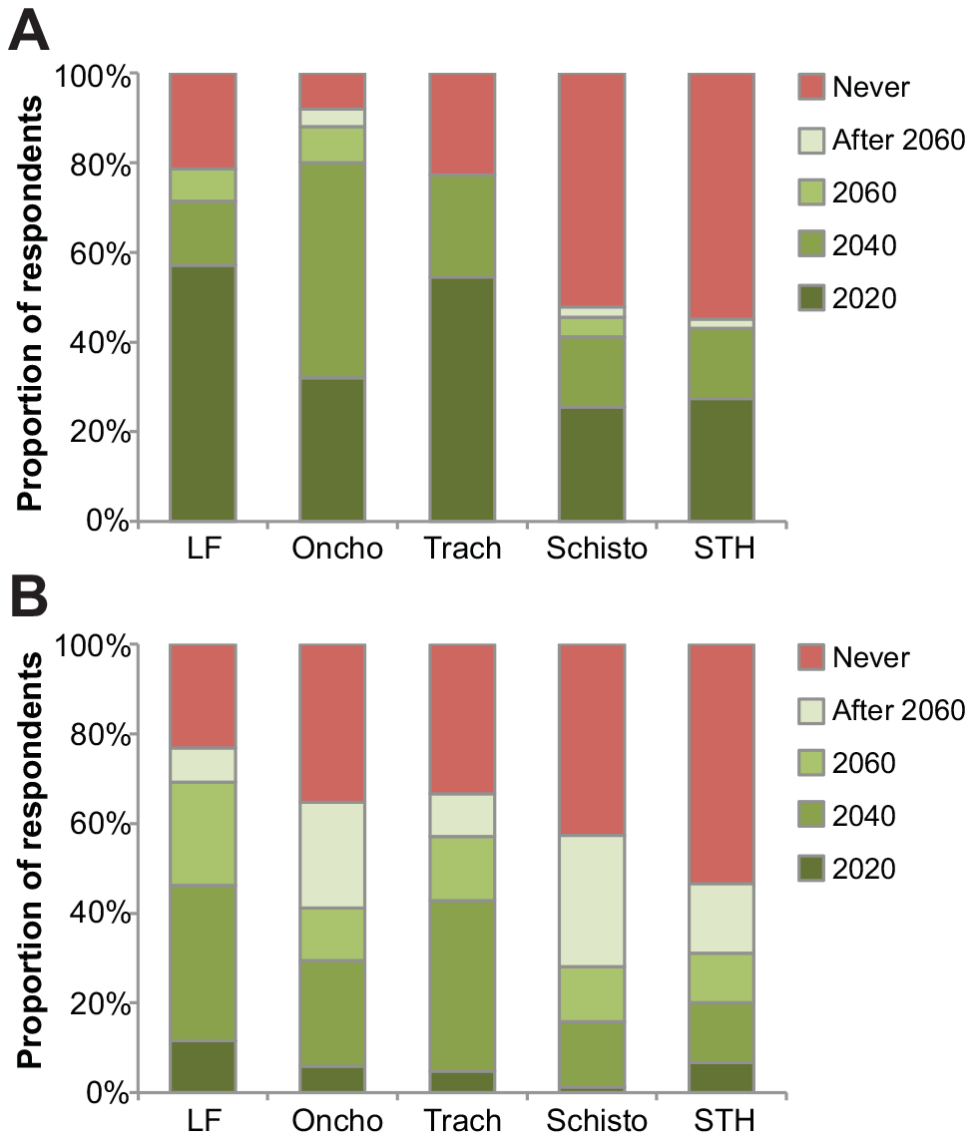
Timeline for elimination and eradication for five neglected tropical diseases. Beliefs regarding the earliest time at which (A) local elimination and (B) global eradication could be achieved in a district if repeated mass drug administrations began in 2011. LF = lymphatic filariasis, Oncho = onchocerciasis, Trach = trachoma, Schisto = schistosomiasis, STHs = soil-transmitted helminths.

We asked when local elimination of a district would occur if repeated MDAs were started immediately (i.e., the year 2011). Respondents were most enthusiastic about the ability of repeated MDAs to eliminate onchocerciasis, LF, and trachoma: greater than 70% of respondents for these 3 diseases believed elimination could occur by 2040, and greater than half of LF and trachoma respondents believed elimination could occur as soon as 2020 (Figure 4A and Table S1; *P*<0.001). When asked about the minimum drug coverage thought necessary for elimination, respondents were in general agreement (the average response ranged from 75.6% for onchocerciasis to 79.4% for STHs, *P* = 0.31, Table S1).

We asked whether MDAs could provide indirect treatment effects to untreated individuals. Respondents differed on this topic, with only 9.1% of onchocerciasis respondents believing herd protection played a role for elimination, versus 55% of trachoma respondents (Table 1). If indirect protection did play a role, certain populations could be targeted for mass treatments. Only schistosomiasis and trachoma respondents reached any sort of consensus on the appropriate target population, with 46.2% of schistosomiasis respondents citing school-aged children as the optimal group to target, and 52.9% of trachoma respondents identifying pre-school-aged children (Table 1).

**Table 1:** Responses to questions of target population for mass drug administrations (MDAs).

	Lymphatic Filariasis	Onchocerciasis	Trachoma	Schistosomiasis	Soil-transmitted Helminths	*P*-value†
Elimination possible from indirect effects of MDAs, N (%)	8/26 (30.8%)	2/22 (9.1%)	12/22 (54.6%)	28/85 (32.9%)	16/48 (33.3%)	0.03
Elimination possible by targeting…*						
Pre-school children	2/22 (9.1%)	2/17 (11.8%)	9/17 (52.9%)	11/65 (16.9%)	7/38 (18.4%)	0.006
School children	5/22 (22.7%)	3/17 (17.7%)	6/17 (35.3%)	30/65 (46.2%)	11/38 (29.0%)	0.10
Those with clinical signs	7/22 (31.8%)	3/17 (17.7%)	3/17 (17.7%)	11/65 (16.9%)	6/38 (15.8%)	0.59
Targeting not effective	10/22 (45.5%)	12/17 (70.6%)	3/17 (17.7%)	26/65 (40.0%)	19/38 (50.0%)	0.03
Other	4/22 (18.2%)	2/17 (11.8%)	5/17 (29.4%)	9/65 (13.9%)	5/38 (13.1%)	0.55

* Respondents were allowed to provide more than 1 response; therefore, percentages within an NTD do not sum to 100%.

†Chi square test.

We asked whether emergent drug resistance could limit the potential for MDA programs. Respondents generally believed that drug resistance was problematic both for the targeted NTD and for unrelated other infections, with the only exception being that most (81.8%) of trachoma respondents believed that chlamydial resistance would not be a major concern (Table 2). Most respondents thought that annual mass treatments would result in less or equivalent resistance compared to the same amount of treatments dispensed throughout the year.

**Table 2:** Responses to questions about drug resistance.

	Lymphatic Filariasis	Onchocerciasis	Trachoma	Schistosomiasis	Soil-transmitted Helminths	*P*-value*
Is drug resistance a problem…						
For the NTD?	14/27 (51.9%)	13/20 (65.0%)	4/22 (18.2%)	53/87 (60.9%)	28/46 (60.9%)	0.005
For another infection?	17/24 (70.8%)	11/17 (64.7%)	16/22 (72.7%)	58/82 (70.7%)	32/41 (78.1%)	0.87
Best strategy to minimize resistance						0.14
(1) Annual mass treatment	9/24 (37.5%)	6/16 (37.5%)	14/21 (66.7%)	29/78 (37.2%)	21/42 (50.0%)	
(2) Treatment scattered throughout year	3/24 (12.5%)	1/16 (6.3%)	1/21 (4.8%)	3/78 (3.9%)	5/42 (11.9%)	
No difference between (1) and (2)	12/24 (50.0%)	9/16 (56.3%)	6/21 (28.6%)	46/78 (59.0%)	16/42 (38.1%)	

* Chi square test.

### Eradication

We also elicited the opinions of respondents regarding global eradication. Respondents for LF were most optimistic that eradication could occur (76.9% of respondents thought eradication possible), followed by trachoma (66.7%), onchocerciasis (64.7%), schistosomiasis (57.3%), and STHs (46.7%). When asked how soon eradication could occur, only LF and trachoma respondents thought it realistic that eradication could occur before 2060, with 69.2% and 57.1% believing this to be the case, respectively. In contrast, eradication by 2060 was thought possible by 28.0% of schistosomiasis respondents, 31.1% of STH respondents, and 41.2% of onchocerciasis respondents (*P* = 0.006 comparing distribution of responses across the five NTDs). Not surprisingly, the most common obstacle to eradication listed for all NTDs was lack of resources (Table 3). Of note, onchocerciasis respondents also cited the lack of an effective treatment as an obstacle for onchocerciasis eradication.

**Table 3:** Reasons listed as obstacles to eradication.

	Lymphatic Filariasis	Onchocerciasis	Trachoma	Schistosomiasis	Soil-transmitted Helminths
	(N = 26)	(N = 19)	(N = 22)	(N = 86)	(N = 45)
Lack of resources	11 (42.3%)	7 (36.8%)	8 (36.4%)	39 (45.4%)	14 (31.1%)
Politics/war	5 (19.2%)	2 (10.5%)	1 (4.6%)	11 (12.8%)	8 (17.8%)
Lack of community awareness	4 (15.4%)	0 (0%)	2 (9.1%)	15 (17.4%)	9 (20.0%)
Ineffective treatment	3 (11.5%)	5 (26.3%)	2 (9.1%)	6 (7.0%)	4 (8.9%)
Antimicrobial resistance	0 (0%)	1 (5.3%)	1 (4.6%)	4 (4.7%)	0 (0%)
Other	3 (11.5%)	4 (21.1%)	8 (36.4%)	11 (12.8%)	10 (22.2%)

*P* = 0.22 comparing the distribution of responses between the five neglected tropical diseases.

### Supplementary analyses

In this report, responses from experts in hookworm, roundworm, and whipworm were generally consistent. Therefore, we grouped these three infections together as STHs to enhance the sample size of this group. However, we also tabulated responses for each of the individual helminths (Table S2).

## Discussion

In this survey, we found that experts in NTDs were generally optimistic about the prospects of elimination for LF, onchocerciasis, and trachoma, but thought control was a more realistic goal for schistosomiasis and STHs. MDAs were thought to be the most important component of treatment for LF, onchocerciasis, and trachoma, whereas other public health measures were most important for schistosomiasis and STHs. Although most respondents believed that elimination could only occur with MDAs plus other health measures, a sizeable minority of experts believed that elimination of LF, onchocerciasis, or trachoma could occur with MDAs alone.

In theory, each of the five most prevalent NTDs could be eliminated. Elimination signifies a reduction in the incidence of infection to zero within a defined geographic area, which may require continued interventions to prevent re-emergence of infection [6], [7]. Elimination is theoretically feasible for any infection which does not have a non-human vertebrate host and cannot amplify in the environment, and for which there is an effective intervention to interrupt transmission and an adequately sensitive and specific diagnostic test to monitor transmission [6]. Using these criteria, elimination of each of the five most prevalent NTDs is a possibility.

MDA is one intervention that effectively reduces infection. Several studies have demonstrated the theoretical potential for eliminating NTDs with MDAs. For example, mathematical models show that six biannual mass azithromycin distributions at an 80% coverage level could eliminate trachoma, that eight rounds of mass ivermectin at 65% coverage could eliminate LF, and that 25 years of mass ivermectin at 65% coverage could eliminate onchocerciasis [8], [9], [10]. These models have generally demonstrated that the ability for mass treatments to eliminate infection depends on treatment frequency, treatment coverage, drug efficacy, and baseline endemicity [8], [9], [10], [11]. In contrast, mathematical models have demonstrated that elimination of STHs through MDAs will be much more difficult to achieve. According to these models, elimination will be especially challenging for hookworm, since unlike the other STHs the bulk of hookworm eggs are not found in the school-aged children to whom mass deworming efforts are currently targeted [12]. These modeling exercises may have influenced respondents, since schistosomiasis and STH experts were much less likely than experts of the other NTDs to believe that elimination was even a possibility (Figure 4).

In addition to mathematical models, empirical studies have demonstrated the potential for MDAs to eliminate each of the five most prevalent NTDs [4]. Five years of annual diethylcarbamazine and albendazole eliminated LF in a hypoendemic region of Egypt, and nearly eliminated infection in a region with higher prevalence [13]. Periodic mass ivermectin treatments for 13–17 years, along with vector control activities, have resulted in elimination of *Onchocerca volvulus* infection in some areas of Africa and the Americas, although not in others [14], [15], [16], [17]. Two biennial (once every two years) mass azithromycin distributions for trachoma eliminated ocular chlamydia infection in a single hypoendemic village in Tanzania, and six biannual (twice yearly) mass azithromycin treatments eliminated infection in two hyperendemic communities in Ethiopia, although elimination of larger geographical areas has proven more difficult [18], [19], [20], [21]. In a study of eight rounds of annual mass praziquantel plus mebendazole distributions in Cambodia, no schistosomiasis infections were detected in a sample of school-aged children during the fifth to seventh years of the study, though infection did re-emerge in the final year [22]. This same study also found zero *Ascaris* or *Trichuris* infections in several villages after the eight rounds of treatment, suggesting the possibility of eliminating these STHs. Therefore, there is at least some evidence that MDAs could result in elimination of each of the five most prevalent NTDs, except for hookworm. Despite these findings, respondents for schistosomiasis and STHs were pessimistic about the prospects of elimination, and especially pessimistic about the idea of relying only on MDAs for elimination. These beliefs may be based in part on what has been achieved by NTD programs outside of the research setting: while there are success stories for elimination of LF, onchocerciasis, and trachoma in several African, Asian, and Latin American countries that have implemented MDAs, this is not the case for schistosomiasis and STH programs [4].

The experts surveyed in this study did not believe that each of the NTDs would be equally easy to eliminate with mass treatments. Instead, as a group, they generally believed that LF, onchocerciasis, and trachoma would be more likely to be eliminated through MDAs than schistosomiasis or STHs. For these latter two infections, they believed that other health measures were more important than MDAs. Although we did not elicit the specific health measures, other studies have highlighted the importance of improved sanitation and water supply for control of helminthic infections [23], [24]. Consistent with this, many believe that MDAs will not be able to eliminate infection without a concurrent improvement in water supply, sanitation, and hygiene education [25], [26].

The optimal level of drug coverage during MDAs is unclear. Although universal coverage would be ideal, attaining high coverage levels requires more resources. The WHO recommends mass antihelminthic coverage levels of ≥75% and mass azithromycin coverage levels exceeding ≥80% of the total population [28], [29]. Survey respondents generally agreed with this recommendation; on average, they thought coverage levels approximating 80% would be necessary in order to achieve local elimination.

Respondents generally did not think that mass treatments targeted to specific high-risk groups could eliminate infection, with more than half of respondents for LF, onchocerciasis, and STHs believing that targeted treatments would not be effective. An exception was trachoma, with more than half of respondents indicating that targeted treatments could eliminate infection. Treatments targeted to high-risk groups have been discussed for some time in the trachoma community, and a recent clinical trial found that treatment of children with azithromycin was effective in reducing chlamydial infection in untreated adults [3]. Respondents who did think targeted treatments could eliminate infection generally thought school-aged children would be the appropriate group to target for schistosomiasis and STHs, while pre-school children would be more appropriate for trachoma. This is not surprising, since the prevalence of helminthic infections generally peaks in the school-age years, whereas trachoma prevalence peaks among pre-school children [30], [31].

Respondents thought that drug resistance was a major challenge for elimination of the NTDs in this survey. Respondents for the four helminthic infections generally thought that resistance was a potential problem for the targeted helminth as well as for unrelated infections, whereas respondents for trachoma felt that resistance was more problematic for non-chlamydial infections. That resistance was thought to be such an important limitation for elimination is notable, since there is currently no evidence for widespread antihelminthic or antibiotic resistance in communities treated with MDAs [32], [33]. This caution may in part be due to the global health community's previous experience with malaria, in which resistance was found to have emerged after the mass distribution of medicated salts [34].

Respondents generally thought that MDA was one of the most important strategies for elimination. Thus, this survey offers indirect support for integrating MDAs in areas with overlap of NTDs. Large areas of the developing world are thought to be affected with two or more NTDs requiring MDAs [27]. Integrated delivery of medications every 6 to 12 months could efficiently provide treatment for several diseases at once. Integrated delivery should save financial resources, and thus address the most important obstacle to eradication identified by the survey respondents. Moreover, combining separate drug campaigns into a single effort would be much more convenient for community members, since they would be required to miss less time from work activities. As a result, it is possible that a higher proportion of the community would be treated in a single campaign than would have been treated with multiple separate campaigns. This could be an important strategy to achieve the 80% coverage that survey respondents thought would be necessary in order for elimination to be achieved. Targeting MDAs to smaller subsets of the population (e.g., children) might provide further efficiencies. Unfortunately, there was no consensus across the five NTDs for any such subpopulation; trachoma researchers tended to think that targeting pre-school children would be most effective, whereas schistosomiasis experts generally thought school-aged children would be the optimal group to target.

There are several limitations to this survey. First, we did not ask respondents whether they had clinical experience in the field with control programs. However, we attempted to restrict the survey only to experts in the field by sending survey invitations to corresponding authors in peer-reviewed journals. We excluded basic science articles as we were concerned that the concepts of elimination, eradication, and MDAs might be outside a basic scientist's area of expertise. Furthermore, we did hear by email from several potential respondents who did not complete the survey because they did not feel qualified to do so (e.g., basic scientists, economists, etc.). Given the general difficulty in achieving high response rates to surveys, it is unlikely that individuals without first-hand clinical knowledge of an NTD would have much incentive to complete the survey. Second, the survey did not expressly seek out program workers because we were unaware of any forum from which we could obtain contact information. We acknowledge that the opinions of program workers carry special value, and that these opinions may be different from those of academics. Third, a short survey cannot capture the complexity of each of these NTDs, and forces each of the diseases into a similar narrow framework, which may not be appropriate. For example, we used the word “infection” and not “disease” throughout the survey, which may be less relevant for certain parasitic diseases such as the STHs. We asked only general questions, without allowing any nuance in terms of the prevalence of infection or public health infrastructure. We did not ask about the role of post-treatment re-infection or rebound morbidity in thwarting control and elimination efforts. Differences in interpretation of the questions may have increased the statistical noise of the survey. These weaknesses aside, this study nonetheless provides an overview of the opinions of NTD experts, and allows comparison of major differences between the five most common NTDs.

The results of this survey provide a snapshot of the opinions of researchers with regards to elimination and eradication of the most prevalent NTDs. Respondents thought that elimination of LF, onchocerciasis, and trachoma was feasible. MDAs were viewed as the most important strategy for elimination for most of the diseases, although most respondents also thought other health measures and community education would be important for elimination. The results of this survey may be helpful when considering integration of mass treatments for multiple NTDs.

## Supporting Information

Table S1 **The role of mass drug administrations for five neglected tropical diseases.** Respondents identified the neglected tropical disease with which they were most familiar and answered several questions. This table shows the exact numbers of responses for each of the diseases, as described in the text and shown in Figures 1,2, and 4.
(DOCX)

Table S2 **Differences in opinions between respondents for the three soil-transmitted helminths.** For the primary analysis, the three soil-transmitted helminths were combined into a single group. In contrast, this table shows a summary of the responses for each of the questions, stratified by helminthic organism.
(DOCX)

Text S1 **The text of the questionnaire that was answered by respondents.** The questionnaire was distributed online through surveymonkey.com (Palo Alto, CA, USA).
(DOC)
