# Supplementary material for: Elimination and Eradication of Neglected Tropical Diseases with Mass Drug Administrations: A Survey of Experts
Source: PLoS Negl Trop Dis. 2013 Dec 5;7(12):e2562. doi: 10.1371/journal.pntd.0002562 (PMC3855072; doi:10.1371/journal.pntd.0002562)
Supplement: Table S1 — The role of mass drug administrations for five neglected tropical diseases. Respondents identified the neglected tropical disease with which they were most familiar and answered several questions. This table shows the exact numbers of responses for each of the diseases, as described in the text and shown in Figures 1,2, and 4. (DOCX) [file pntd.0002562.s001.docx]

**Supplementary Table 1.**

|  | Lymphatic Filariasis | Onchocerciasis | Trachoma | Schistosomiasis | Soil-transmitted Helminths |
| --- | --- | --- | --- | --- | --- |
| Goal of mass drug administrations (MDAs) (Fig 1) | N=32 | N=27 | N=22 | N=97 | N=55 |
| Control | 4 (13%) | 11 (41%) | 10 (45%) | 75 (77%) | 43 (78%) |
| Elimination | 14 (44%) | 13 (48%) | 9 (41%) | 19 (20%) | 8 (15%) |
| Eradication | 14 (44%) | 3 (11%) | 3 (14%) | 3 (3%) | 4 (7%) |
|  |  |  |  |  |  |
| Elimination possible through… (Fig 2) | N=29 | N=27 | N=20 | N=93 | N=52 |
| MDA Alone | 6 (21%) | 7 (26%) | 4 (20%) | 1 (1%) | 1 (2%) |
| MDA+ | 14 (48%) | 14 (52%) | 13 (65%) | 59 (63%) | 30 (58%) |
| Other Measures Alone | 0 (0%) | 0 (0%) | 1 (5%) | 7 (8%) | 5 (10%) |
| New Test/Tool | 8 (28%) | 5 (19%) | 1 (5%) | 22 (24%) | 10 (19%) |
| Elimination not possible | 1 (3%) | 1 (4%) | 1 (5%) | 4 (4%) | 6 (12%) |
|  |  |  |  |  |  |
| Repeated MDAs alone could result in elimination by… (Fig 4A) | N=28 | N=25 | N=22 | N=90 | N=51 |
| 2020 | 16 (57%) | 8 (32%) | 12 (55%) | 23 (26%) | 14 (27%) |
| 2040 | 4 (14%) | 12 (48%) | 5 (23%) | 14 (16%) | 8 (16%) |
| 2060 | 2 (7%) | 2 (8%) | 0 (0%) | 4 (4%) | 0 (0%) |
| After 2060 | 0 (0%) | 1 (4%) | 0 (0%) | 2 (2%) | 1 (2%) |
| Never | 6 (21%) | 2 (8%) | 5 (23%) | 47 (52%) | 28 (55%) |
|  |  |  |  |  |  |
| Minimum drug coverage for elimination | N=26 | N=18 | N=21 | N=76 | N=44 |
| Mean  (95% Confidence Interval) | 77.3%  (71.2-83.5%) | 75.6%  (70.6-80.5%) | 78.6%  (71.4-85.7%) | 78.7%  (73.6-83.7%) | 79.4%  (73.3-85.5%) |
|  |  |  |  |  |  |
| Repeated MDAs alone could result in eradication by…(Figure 4B) | N=26 | N=17 | N=21 | N=82 | N=45 |
| 2020 | 3 (12%) | 1 (6%) | 1 (5%) | 1 (1%) | 3 (7%) |
| 2040 | 9 (35%) | 4 (24%) | 8 (38%) | 12 (15%) | 6 (13%) |
| 2060 | 6 (23%) | 2 (12%) | 3 (14%) | 10 (12%) | 5 (11%) |
| After 2060 | 2 (8%) | 4 (24%) | 2 (10%) | 24 (29%) | 7 (16%) |
| Never | 6 (23%) | 6 (35%) | 7 (33%) | 35 (43%) | 24 (53%) |
|  |  |  |  |  |  |
| Academic degree of respondent | N=27 | N=20 | N=22 | N=86 | N=46 |
| MPH or other Masters | 1 (4%) | 1 (5%) | 1 (5%) | 3 (3%) | 2 (4%) |
| MD | 3 (11%) | 3 (15%) | 5 (23%) | 12 (14%) | 10 (22%) |
| PhD | 18 (67%) | 13 (65%) | 11 (50%) | 60 (70%) | 26 (57%) |
| MD, PhD | 5 (19%) | 3 (15%) | 5 (23%) | 11 (13%) | 8 (17%) |
|  |  |  |  |  |  |
| Region of Research* | N=27 | N=20 | N=22 | N=86 | N=46 |
| Africa | 12 (44%) | 14 (70%) | 14 (64%) | 46 (53%) | 13 (28%) |
| Middle East | 2 (7%) | 1 (5%) | 1 (5%) | 6 (7%) | 2 (4%) |
| East Asia | 10 (37%) | 0 (0%) | 1 (5%) | 21 (24%) | 10 (22%) |
| South Asia | 5 (19%) | 0 (0%) | 1 (5%) | 9 (10%) | 8 (17%) |
| Central Asia | 0 (0%) | 0 (0%) | 0 (0%) | 4 (5%) | 3 (7%) |
| Australia | 1 (4%) | 0 (0%) | 2 (9%) | 1 (1%) | 2 (4%) |
| Europe | 4 (15%) | 1 (5%) | 1 (5%) | 9 (10%) | 3 (7%) |
| North America | 5 (19%) | 3 (15%) | 3 (14%) | 5 (6%) | 2 (4%) |
| South America | 4 (15%) | 7 (35%) | 4 (18%) | 22 (26%) | 15 (33%) |

* Some respondents worked in more than one region, so numbers do not sum to 100%
