# Supplementary material for: Elimination and Eradication of Neglected Tropical Diseases with Mass Drug Administrations: A Survey of Experts
Source: PLoS Negl Trop Dis. 2013 Dec 5;7(12):e2562. doi: 10.1371/journal.pntd.0002562 (PMC3855072; doi:10.1371/journal.pntd.0002562)
Supplement: Table S2 — Differences in opinions between respondents for the three soil-transmitted helminths. For the primary analysis, the three soil-transmitted helminths were combined into a single group. In contrast, this table shows a summary of the responses for each of the questions, stratified by helminthic organism. (DOCX) [file pntd.0002562.s002.docx]

**Supplementary Table 2.**

|  | **Hookworm** | **Roundworm** | **Whipworm** |
| --- | --- | --- | --- |
| Goal of MDAs | N=32 | N=18 | N=5 |
| Control | 26 (81.3%) | 14 (77.8%) | 3 (60%) |
| Elimination | 5 (16%) | 3 (17%) | 0 (0%) |
| Eradication | 1 (3%) | 1 (6%) | 2 (40%) |
|  |  |  |  |
| Elimination possible through… | N=30 | N=18 | N=4 |
| MDA Alone | 1 (3%) | 0 (0%) | 0 (0%) |
| MDA+ | 17 (57%) | 10 (56%) | 3 (75%) |
| Other Measures Alone | 1 (3%) | 4 (22%) | 0 (0%) |
| New Test/Tool | 7 (23%) | 3 (17%) | 0 (0%) |
| Elimination not possible | 4 (13%) | 1 (6%) | 1 (25%) |
|  |  |  |  |
| Repeated MDAs alone could result in elimination by… | N=30 | N=17 | N=4 |
| 2020 | 7 (23%) | 5 (29%) | 2 (50%) |
| 2040 | 6 (20%) | 2 (12%) | 0 (0%) |
| 2060 | 0 (0%) | 0 (0%) | 0 (0%) |
| After 2060 | 1 (3%) | 0 (0%) | 0 (0%) |
| Never | 16 (53%) | 10 (59%) | 2 (50%) |
|  |  |  |  |
| Minimum drug coverage for elimination | N=25 | N=15 | N=4 |
| Mean (95% CI)* | 81.2% (72.8-88.4%) | 80.1% (67.6-88.5%) | 65.0% (45.0-90.0%) |
|  |  |  |  |
| Elimination possible from indirect effects of MDAs, N (%) | N=27 | N=17 | N=4 |
| Yes | 8 (30%) | 5 (29%) | 3 (75%) |
| No | 29 (70%) | 12 (71%) | 1 (25%) |
|  |  |  |  |
| Elimination possible by targeting… | N=23 | N=12 | N=3 |
| Pre-school children | 2 (9%) | 3 (25%) | 2 (67%) |
| School children | 4 (17%) | 4 (33%) | 3 (100%) |
| Those with clinical signs | 5 (22%) | 0 (0%) | 1 (33%) |
| Targeting not effective | 14 (61%) | 5 (42%) | 0 (0%) |
| Other | 3 (13%) | 2 (17%) | 0 (0%) |
|  |  |  |  |
| Is drug resistance a problem for the NTD? | N=27 | N=15 | N=4 |
| Yes | 18 (67%) | 7 (47%) | 3 (75%) |
| No | 9 (33%) | 8 (53%) | 1 (25%) |
|  |  |  |  |
| Is drug resistance a problem for another infection? | N=26 | N=12 | N=3 |
| Yes | 22 (85%) | 8 (67%) | 2 (67%) |
| No | 4 (15%) | 4 (33%) | 1 (33%) |
|  |  |  |  |
| Best strategy to minimize resistance | N=23 | N=15 | N=4 |
| (1) Annual mass treatment | 13 (57%) | 6 (40%) | 2 (50%) |
| (2) Treatment scattered throughout year | 2 (9%) | 2(13%) | 1 (25% |
| No difference between (1) and (2) | 8 (35%) | 7 (47%) | 1 (25%) |
|  |  |  |  |
| Repeated MDAs alone could result in eradication by… | N=26 | N=15 | N=4 |
| 2020 | 1 (4%) | 1 (7%) | 1 (25%) |
| 2040 | 3 (12%) | 3 (20%) | 0 (0%) |
| 2060 | 3 (12%) | 2 (13%) | 0 (0%) |
| After 2060 | 5 (19%) | 2 (13%) | 0 (0%) |
| Never | 14 (54%) | 7 (47%) | 3 (75%) |
|  |  |  |  |
| Obstacles to Eradication | N=27 | N=14 | N=4 |
| Lack of resources | 10 (37%) | 3 (21%) | 1 (25%) |
| Politics/war | 3 (11%) | 4 (29%) | 1 (25%) |
| Lack of community awareness | 4 (15%) | 3 (21%) | 2 (50%) |
| Ineffective treatment | 3 (11%) | 1 (7%) | 0 (0%) |
| Antimicrobial resistance | 0 (0%) | 0 (0%) | 0 (0%) |
| Other | 7 (26%) | 3 (21%) | 0 (0%) |

*Bias-corrected, 95% bootstrapped confidence interval
