# Supplementary material for: Elimination and Eradication of Neglected Tropical Diseases with Mass Drug Administrations: A Survey of Experts
Source: PLoS Negl Trop Dis. 2013 Dec 5;7(12):e2562. doi: 10.1371/journal.pntd.0002562 (PMC3855072; doi:10.1371/journal.pntd.0002562)
Supplement: Text S1 — The text of the questionnaire that was answered by respondents. The questionnaire was distributed online through surveymonkey.com (Palo Alto, CA, USA). (DOC) [file pntd.0002562.s003.doc]

(Note that his header was present on every screen of the survey)


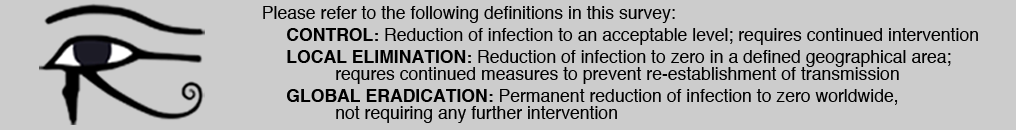


**1. Select the neglected tropical disease for which you have the most expertise or interest.**

1. Hookworm
2. Roundworm (Ascaris)
3. Whipworm (Trichuris)
4. Lymphatic filariasis
5. Onchocerciasis
6. Schistosomiasis
7. Trachoma
8. Chagas Disease (Trypanosoma cruzi)
9. Sleeping Sickness (Trypanosoma brucei)
10. Leprosy
11. Visceral Leishmaniasis
12. Other (Write in box below)

**If answered (a) through (g), then go to Question #2. If answered (h) through (l), then go to Question #3.**

**2. Do mass drug administrations have a role for the treatment of the selected infection?**

1. Yes
2. No

**3. The following diseases are commonly treated with mass drug administrations (MDAs). Even if one of these diseases is not your primary clinical or research interest, we are still interested in your thoughts on mass drug administrations. Please select the disease with which you are most familiar and answer the remaining questions regarding that disease.**

1. Hookworm
2. Roundworm (Ascaris)
3. Whipworm (Trichuris)
4. Lymphatic filariasis
5. Onchocerciasis
6. Schistosomiasis
7. Trachoma
8. No familiarity with any of these NTDs
9. Other disease where mass drug administrations useful (Write in box below)

**Please answer the remaining questions in the survey as they pertain to the disease you selected. If you have expertise in more than 1 disease, you will be able to answer questions about other diseases later in the survey.**

**Please note that the definitions used in this survey will be shown on each page in the grey box above.**

**4. What is the most important goal of mass drug administrations (MDAs) for the selected neglected tropical disease?**

1. CONTROL (reduction of infection to an acceptable level; requires continued intervention)
2. LOCAL ELIMINATION (reduction of infection to zero in a defined geographical area; requires continued measures to prevent re-establishment of transmission)
3. GLOBAL ERADICATION (permanent reduction of infection to zero worldwide, not requiring any further intervention)

**5. Under what circumstances do you think LOCAL ELIMINATION of the selected infection is possible?**

1. Mass drug administrations alone could eliminate infection (using currently available drugs)
2. Mass drug administrations plus other health measures could eliminate infection (using currently available drugs and health measures)
3. Other health measures alone could eliminate infection (using currently available health measures)
4. Elimination is not possible with currently available drugs or health measures, but a new diagnostic test or interventional tool (e.g., vaccine, medication) could

eliminate infection

1. Elimination not possible (with current strategies, nor with potential new diagnostic or interventional tools)

**6. Please rank the following from most important to least important for LOCAL ELIMINATION of the selected infection.**

**1 [Most] 2 3 4 5 6 [Less]**

1. Programmatic mass drug administrations
2. Programmatic health measures (e.g., sanitation, hygiene)
3. Programmatic community participation/education
4. Programmatic vector control (flies, snails, mosquitos)
5. Non-programmatic measures (i.e., secular trend)
6. Development of new programmatic diagnostic test or intervention (e.g., vaccine, medication)

**7. If repeated mass drug administrations were started now in a region (e.g., an area with at least 1 million people) that was severely affected with the selected infection, by what year would LOCAL ELIMINATION most likely occur in the region? (Assume that other public health interventions for the infection remain unchanged.)**

1. 2015
2. 2020
3. 2030
4. 2040
5. 2050
6. 2060
7. After 2060
8. MDAs cannot eliminate infection

**8. How sure are you of this estimate regarding LOCAL ELIMINATION?**

1. Very certain (within a range of 5 years)
2. Fairly certain (within a range of 10 years)
3. Somewhat certain (within a range of 20 years)
4. Not very certain (within a range of 30 years)
5. Not certain at all (within a range of 40 years)

**9. What is the minimum percentage of the population that must be treated during periodically repeated mass drug administrations in order to LOCALLY ELIMINATE the selected infection? (Please enter a number between 0 and 100)**

**10. Could the selected disease be LOCALLY ELIMINATED by targeting only a portion of the community (i.e., indirect protection of untreated individuals)?**

1. Yes
2. No

**11. If so, what is the ideal target population for ELIMINATION of the selected infection? (Select all that apply.)**

1. Pre-school children (approximately ages 0-5 years)
2. School-aged children (approximately ages 6-15 years)
3. Individuals with clinical signs of disease
4. N/A (Targeting a specific population will not be effective)
5. Other

**12. Is drug resistance a challenge to ELIMINATION / ERADICATION...**

...for the selected neglected tropical disease itself?

1. Yes
2. No

...for infections unrelated to the selected neglected tropical disease?

1. Yes
2. No

**13. Please select which situation below would MINIMIZE drug resistance for the selected infection:**

1. Repeated annual mass treatment of the entire community at the same single time point each year, with very high drug coverage
2. Use of the same number of doses of drug as above, but distributed evenly throughout the year
3. Both options above will result in similar amounts of resistance

**14. When will GLOBAL ERADICATION of the selected infection occur?**

1. 2015
2. 2020
3. 2030
4. 2040
5. 2050
6. 2060
7. After 2060
8. Eradication is not possible

**15. How sure are you of this estimate regarding GLOBAL ERADICATION?**

1. Very certain (within a range of 5 years)
2. Fairly certain (within a range of 10 years)
3. Somewhat certain (within a range of 20 years)
4. Not very certain (within a range of 30 years)
5. Not certain at all (within a range of 40 years)

**16. What is the biggest obstacle to GLOBALLY ERADICATING the specified infection?**

1. Lack of resources
2. Ineffective treatment
3. Antimicrobial resistance
4. Community awareness/involvement
5. Politics/war
6. Other

**17. Please select your degree. (Select all that apply.)**

1. MD or equivalent
2. PhD or equivalent
3. MPH or equivalent
4. Other Masters
5. Bachelors or equivalent
6. Other
7. Other (please specify)

**18. Where do you conduct research for the selected neglected tropical disease? (Select all that apply.)**

1. Sub-saharan Africa
2. North Africa/Middle East
3. East/Southeast Asia
4. South Asia
5. Central Asia
6. Australia
7. Europe
8. South America
9. North America

**19. Do you have expertise in another neglected tropical disease?**

1. Yes, and I'm willing to answer the same questions about that disease
2. Yes, but I don't have time for any more questions
3. No

**If answered (a), then go to Question #20. If answered (b) or (c), then finish survey.**

**20. Please select a second neglected tropical disease and answer the following survey questions in regards to the selected disease.**

1. Hookworm
2. Roundworm (Ascaris)
3. Whipworm (Trichuris)
4. Lymphatic filariasis
5. Onchocerciasis
6. Schistosomiasis
7. Trachoma
8. Other (Write in box below)
9. Other

**Then continues with survey from Question #4 above.**
